# Tuina therapy promotes behavioral improvement and brain plasticity in rats with peripheral nerve injury and repair

**DOI:** 10.1002/brb3.3174

**Published:** 2023-07-31

**Authors:** Shu‐Jie Ma, Jun‐Peng Zhang, Xu‐Yun Hua, Jia‐Jia Wu, Mou‐Xiong Zheng, Jian‐Guang Xu

**Affiliations:** ^1^ Department of Traditional Chinese Rehabilitation Medicine The Second Rehabilitation Hospital of Shanghai Shanghai China; ^2^ Engineering Research Center of Traditional Chinese Medicine Intelligent Rehabilitation Ministry of Education Shanghai China; ^3^ School of Rehabilitation Science Shanghai University of Traditional Chinese Medicine Shanghai China; ^4^ Department of Traumatology and Orthopedics, Yueyang Hospital Shanghai University of Traditional Chinese Medicine Shanghai China; ^5^ Department of Rehabilitation Medicine, Yueyang Hospital Shanghai University of Traditional Chinese Medicine Shanghai China

**Keywords:** cortical plasticity, functional magnetic resonance imaging, manipulation, massage, peripheral nerve injury, tuina

## Abstract

**Introduction:**

Tuina is currently one of the popular complementary and alternative methods of rehabilitation therapy. Tuina can improve patients' pain and mobility function. However, the underlying physiological mechanism remains largely unknown, which might limit its further popularization in clinical practice. The aim of this study is to explore the short‐term and long‐term changes in brain functional activity following Tuina intervention for peripheral nerve injury repair.

**Methods:**

A total of 16 rats were equally divided into the intervention group and the control group. Rats in the intervention group received Tuina therapy applying on the gastrocnemius muscle of the right side for 4 months following sciatic nerve transection and immediate repair, while the control group received nerve transection and repair only. The block‐design functional magnetic resonance imaging scan was applied in both groups at 1 and 4 months after the surgery. During the scan, both the injured and intact hindpaw was electrically stimulated according to a “boxcar” paradigm.

**Results:**

When stimulating the intact hindpaw, the intervention group exhibited significantly lower activation in the somatosensory area, limbic/paralimbic areas, pain‐regulation areas, and basal ganglia compared to the control group, with only the prefrontal area showing higher activation. After 4 months of sciatic nerve injury, the control group exhibited decreased motor cortex activity compared to the activity observed at 1 month, and the intervention group demonstrated stronger bilateral motor cortex activity compared to the control group.

**Conclusion:**

Tuina therapy on the gastrocnemius muscle of rats with sciatic nerve injury can effectively alleviate pain and maintain the motor function of the affected limb. In addition, Tuina therapy reduced the activation level of pain‐related brain regions and inhibited the decreased activity of the motor cortex caused by nerve injury, reflecting the impact of peripheral stimulation on brain plasticity.

## Graphical abstract

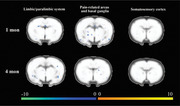

## 1 INTRODUCTION

Tuina, as the traditional Chinese massage, serves as a complementary and alternative method in the rehabilitation therapy. It consists of many classic manipulation methods which are grouped into four distinct categories: pushing‐rolling, squeezing/pressing, moving joints, and vibrating (Fang et al., 2018). Accumulating literature have reported its application in multiple diseases including back pain, cervical vertigo, insomnia, headaches, and hypertension (F. Huang et al., 2020; T. Li et al., 2021; Nie et al., 2019; X. Yang et al., 2014; Y. Zhang et al., 2020). According to existing clinical trials, application of Tuina in several diseases has been proven to be inspiringly effective, especially in pain relieving (Happe et al., 2016; Sousa, Coimbra, et al., 2015; Tang et al., 2016; Wang, 2012). However, the underlying physiological mechanism still remains largely unaddressed.

Early clinical studies have shown that Tuina therapy can effectively alleviate chronic neck pain and exhibit long‐term effects after 12 weeks (Pach et al., 2018). In addition, the combination of Tuina therapy and traditional Chinese exercise is also beneficial in reducing pain and improving disability (Zhou et al., 2022). Studies have shown that Tuina therapy intervention in rats with sciatic nerve pathological pain reduced the spinal dorsal horn C‐fiber response, suggesting that the analgesic effect of Tuina therapy is related to the increase in pain threshold of C‐fiber‐induced field potential of ipsilateral and contralateral nerves (Jiang et al., 2016). The analgesic mechanism of Tuina therapy in peripheral pathological pain is mainly manifested by regulating the TLR4 pathway and miRNA to inhibit peripheral inflammation, regulating ion channels, inhibiting the activation of neuroglia cells, and regulating brain function changes. Tuina therapy has analgesic effects by acting on different levels of targets and is an effective treatment for peripheral neuropathic pain (Z. F. Liu et al., 2022).

A systematic review of the effects of Tuina therapy on poststroke sequelae improvement showed that, in addition to traditional therapies, therapeutic massage, especially Tuina therapy, has a significant effect on improving the motor function and reducing spasticity of stroke survivors (Cabanas‐Valdes et al., 2021). The use of Tuina has shown positive effects in promoting the recovery of patients' motor function after injury (Kang et al., 2022).

As Tuina is a therapy performed locally, researchers used to focus on the physiological mechanism of its peripheral effects. For example, a previous study revealed that Tuina might decrease the activation level of peripheral nociceptive C‐fiber (Jiang et al., 2016). It reasoned that Tuina may induce long nerve fiber signaling and thereby inhibit transmission of pain signal to the central nervous system by activating inhibitory neurons. Some other researchers suggested that neurotransmitters might also play an important role in the therapeutic effect of Tuina (Guo et al., 2016; S. R. Huang et al, 2003; Sousa, Moreira, et al., 2015). In a biomolecular study,  the gene expression at the point of nerve injury and the myelin integrity was modulated by Tuina, which was related to the functional recovery following peripheral nerve injury repair (T. Lv, Mo, Yu, Zhang, et al., 2020). However, the effect of Tuina on the central nervous system following peripheral nerve injury is rarely referred to.

Peripheral nerve injury is one of the major neuropathies that lead to lifelong disabilities (R. Li et al., 2014). Current therapeutic theory suggests that nerve regeneration and target muscle atrophy are two most important factors that influence functional outcomes (Q. Bao et al., 2021; He et al., 2022; Ruven et al., 2017; Sun et al., 2022; Zainul et al., 2022). Previous studies demonstrated that brain plasticity was also an important factor that involves in the recovery other than peripheral regeneration (Navarro et al., 2007). As more studies regarding to Tuina therapy in the treatment of peripheral nerve injury have been reported, investigations on brain plasticity have become essential for a better understanding of its effects and appropriate application in clinical practice (Z. Liu et al., 2021; T. T. Lv, Mo, Yu, Shao, et al., 2020).

Functional magnetic resonance imaging (fMRI) is a safe and noninvasive method widely used in neuroscience research (Fox, 2018; Rocca & Filippi, 2006). It is often used to explore the central nervous system mechanisms of both central nervous system diseases (Shan et al., 2023) and other peripheral diseases (Bhat et al., 2017; Xing et al., 2020, 2021).

Previous fMRI studies have found that the anterior cingulate cortex (ACC), insular cortex, and primary and secondary somatosensory cortices (S1 and S2) are involved in the complex experience of pain (H. Li et al., 2022). In our previous study on electroacupuncture intervention for knee osteoarthritis, we observed that electroacupuncture reduced the centrality and nodal efficiency of the right ACC, which is a central component of the reward circuitry (J. P. Zhang et al., 2023). This inhibition of activity in the reward circuitry contributed to pain suppression and reduced the transmission of pain signals from the source to the central nervous system. Currently, research on the brain mechanisms of pain has expanded from the study of a single brain region to the level of functional connectivity and functional networks (B. B. Bao et al., 2022). Researchers hope to use changes in the cortical function and plasticity as prognostic factors related to pain (Jenkins et al., 2023).

In the present study, the fMRI study was used to describe the alteration of brain activity induced by Tuina therapy as a rehabilitation treatment following peripheral nerve injury repair. The Tuina therapy was applied on the gastrocnemius muscle of the right side for 4 months following sciatic nerve transection and immediate repair, while the fMRI scan and the behavior test were applied at the first and fourth months after the surgery. The aim of this study is to explore the short‐ and long‐term changes in brain functional activity induced by Tuina therapy in peripheral nerve stimulation tasks, to understand the effects of Tuina on pain‐related circuits and motor‐related cortex function regulation, and to further understand the central neural plasticity mechanisms of Tuina in the recovery of peripheral nerve injury.

## 2 MATERIALS AND METHODS

### 2.1 Animals

A total of 16 male Sprague‐Dawley rats weighted from 200 to 250 g and aged from 6 to 8 weeks were used in the study. These rats were randomly divided into the experimental and control groups (eight rats in each group). All of the rats were obtained from Shanghai Slack Laboratory Animal Company. They were housed under a condition of 12 h dark/12 h light cycle, with unrestricted food and water supply. They were kept for 1 week before any intervention started.

### 2.2 Peripheral nerve injury model

**FIGURE 1:**
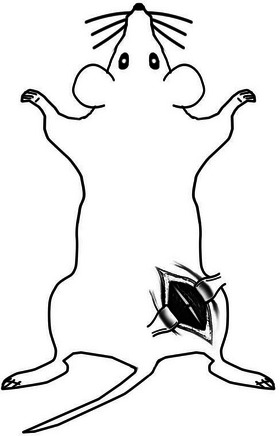
Peripheral nerve injury model. The peripheral nerve injury model was established by cutting and immediately repairing the sciatic nerve of the rat.

The peripheral nerve injury model was established by cutting and immediately repairing the sciatic nerve (Figure 1). Specifically, after an intraperitoneal injection of 0.2% pentobarbital, the sciatic nerve on the right side was completely transected at midthigh level. Epineurium of the sciatic nerve was immediately anastomosed with 12‐0 sutures (Ren et al., 2016). During the surgery, bipolar coagulation was utilized for hemostasis, in order to reduce postsurgical mortality. The incision area was treated with antibiotic powder and sealed with Michel clips.

### 2.3 Tuina therapy

**FIGURE 2:**
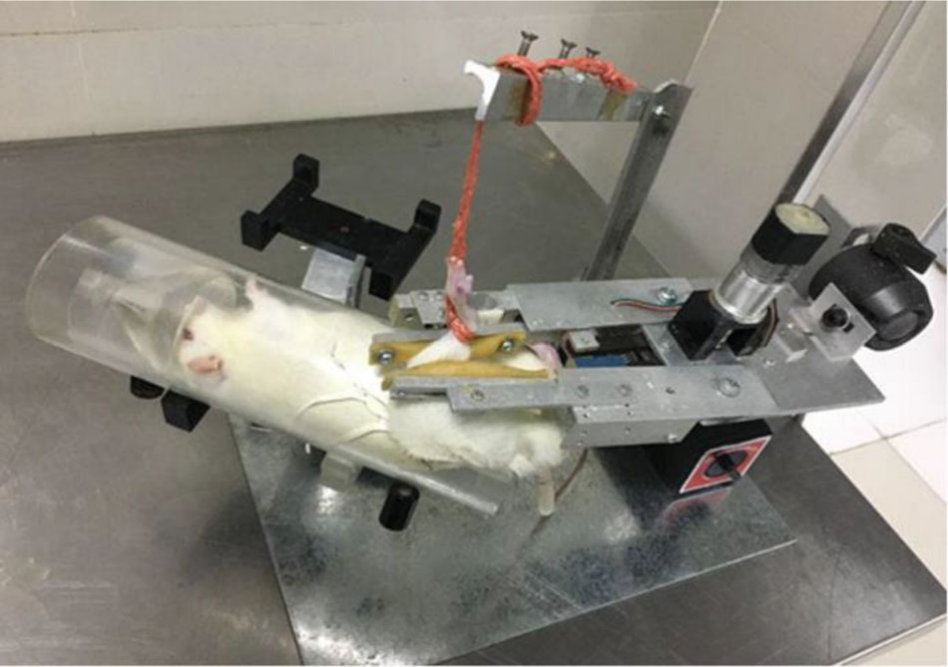
Illustration of the Tuina manipulation emulator. The photograph shows the Tuina manipulation emulator and its application on the rat. The emulator's key structural elements consisted of a controller, a microengine, a pair of mobile arms, several pressure transducers, and a silicone tube. The mobile arms with periodic movement were designed to simulate manipulation by human hands. The intervention parameters including pressure, frequency, and duration were set before the procedure started.

A customized Tuina manipulation emulator (patent No. ZL201420482075.3, State Intellectual Property Office) was used in the present study. The emulator's key structural elements consisted of a controller, a microengine, a pair of mobile arms, several pressure transducers, and a silicone tube (Figure 2). The mobile arms with periodic movement were designed to simulate manipulation by human hands. The intervention parameters including pressure, frequency, and duration were set before the procedure started. During the procedure, the pressure could be regulated by the tension adjusting screw, the tension spring, and sensor be monitored on the screen, and the frequency be controlled by the adjusting knob. The emulator was used to simulate twirling and kneading manipulation on the gastrocnemius muscle of the injured hindlimb. The force was set at 0.45 N, and the frequency was 60 times/min, 10 min a day. Tuina manipulation started on the seventh postoperative day.

To guarantee the consistency of models, one specific technician performed all the surgery and Tuina intervention.

### 2.4 Behavior tests

#### 2.4.1 Thermal withdrawal latency

Thermal withdrawal latency (TWL) of the injured hindpaw was measured using the Hargreaves method for mice and rats by a plantar test apparatus (IITC Life Science Inc., Woodland Hills, CA) every 1 month after the surgery. In the test, the rat was placed quietly on the plexiglass platform for 30 min to adapt to the environment. Thermal stimulation was given by a moveable thermal radiant heat source to the lateral plantar surface of the right (injured) hindpaw. The time from the onset of heat to the withdrawal of the hindpaw was recorded as the TWL. In order to avoid tissue injury, a maximal automatic cutoff latency was set to 20 s. Each test was repeated three times with at least a 5‐min interval between two tests.

#### 2.4.2 CatWalk gait analysis

The animal gait analysis system is a popular tool for the quantitative assessment of footsteps and natural gait in animal models of nerve disease, neurotic atrophy, nerve trauma, and pain symptom groups. In the present study, the Catwalk XT animal gait analysis system (Noldus Information Technology, Wageningen, The Netherlands) was used to evaluate motor function in rats. The system included a 1.5‐m long walking platform with a glass walkway at the bottom. The light‐emitting diode emitted light scattering into the glass plate of the walkway. When the rat passed the walkway, the footprints were captured by a high‐speed camera placed underneath and the speed and intensity were recorded.

Two weeks before the surgery, adaptive training was carried out every day in the morning and afternoon. During the training, 12 g/day of food was given daily to cause slight starvation. An eligible training requires at least three consecutive uninterrupted runs. The conditional parameters for a qualified run were as follows: passing time 1.00–8.00 s, speed variation rate less than 60%. The gait CatWalk analysis was carried out every month for 4 months after the surgery. The maximum contact mean intensity (MCMI), stride length (SL), and swing speed (SS) were recorded.

#### 2.4.3 Statistical analysis of behavioral tests

The behavioral results were expressed as mean ± standard deviation (mean ± *SD*) and analyzed using SPSS 22.0 software package (SPSS Inc., Chicago, IL). A multifactor repeated measures analysis of variance was performed to compare the behavioral data of the control group and intervention group at the four time points for TWL, MCMI, SL, and SS. Correlation analysis was performed between the behavioral data and fMRI results separately. Values of *p* < .05 were considered to be statistically significant.

### 2.5 Functional MRI scan

All the fMRI scans were performed in a 7.0‐T horizontal‐bore Bruker scanner (Bruker Corporation, Mannheim, Germany) with a gradient system of 116 mm inner diameter, of which the maximum gradient strength achieved 400 mT/m. A single transmit and receive surface coil which involved a single copper‐wire loop was utilized. During the scan, rats were anesthetized by sevoflurane, fixed on the scanner, and supported with an artificial ventilator. Their heartbeat and respiratory rate were monitored during the scans. The fMRI scans were performed at the first and fourth postoperative months. The parameters of the functional image were listed as followed, an interleaved single‐shot Echo‐planar imaging (EPI) sequence, flip angle 90°, slice thickness 0.5 mm, repetition time 3000 ms, echo time 20 ms, number of averages 1, Field of view (FOV) 32 × 32 mm with 64 × 64 points. The localization scan and the functional imaging covered a limited area that centered on the bregma point.

#### 2.5.1 Stimulation tasks

A dummy scan lasted for 8 s in the time series was applied before any task was performed. The dummy scan was performed for magnetic equilibrium and the initial 8 s of data would not be included in the analysis. A “boxcar” model for the stimulation paradigm was used, which sequentially contained an epoch of ON and an epoch of OFF. Both “ON” and “OFF” epochs lasted for 30 s and these two epochs formed one cycle. In order to acquire significantly positive results, a total of eight cycles were arranged in one stimulation session. During each session, only one side hindpaw was stimulated. The electrical stimulation was applied with needles inserted beneath the skin of each hindpaw. One needle electrode was located between the first and second digits, while the other one was located between the third and fourth digits. In order to avoid habituation of sensory stimulation, the stimulus was performed in a pseudorandom pattern. The fMRI scans were performed at the first and fourth months after surgery.

#### 2.5.2 Data processing and statistical analysis

The preprocessing and statistical analysis were mainly performed by a toolbox, SPM 8 (Statistical Parametric Mapping 8, http://www.fil.ion.ucl.ac.uk/spm/) based on the Matlab platform. First, the Bruker format data were transformed to the Nifti (Neuroimaging Informatics Technology Initiative) format by the Bru2nii toolbox. Then all the images transformed were scaled up by 10 times of the original size, which was close to the size of the human brain. This upscaling procedure only altered the dimension descriptor fields in the file header and enabled possible usage of processing algorithms originally created for the human brain (Pallares et al., 2015; Tambalo et al., 2015). Preprocessing procedures include the following steps: (1) The nonbrain tissue was stripped manually before further analysis; (2) slice timing: it was performed to remove the temporal bias caused by the different slice acquisition time; (3) realign: images were realigned with rigid‐body transformation and the voxel‐misplacement caused by subjects’ slight head motion were corrected. Through this realignment, a mean volume of functional images was generated; (4) normalization: in order to transform the images from the individual space into the standard space, a transformation matrix was estimated utilizing the mean volume and a standard T2‐weighted template. The estimated ind‐std matrix was then written into every functional volume; (5) smooth: finally, the normalized images were smoothed by a kernel of twice of the voxel size (full width at half maximum, FWHM = [6 6 6]).

In the first‐level analysis, a general linear model was determined according to the stimulation paradigm and the parameters were estimated through a Bayesian approach by SPM8. The contrast images containing information on β1–β2 were then acquired. In the second‐level analysis, a two‐sample t‐test was performed between the experimental and the control groups at the same time point. In addition, paired *t*‐tests were conducted to compare the brain activity between the fourth month and the first month within each group. After the *t*‐value map was generated with contrast vectors, the threshold was set as *p* < .05 and the results were then interrogated with the false discovery rate (FDR) correction. The statistical map was visualized by projecting to a standard rodent template. The analysis and results were reported based on the Montreal Neurological Institute (MNI) atlas standard.

## 3 RESULTS

### 3.1 Behavioral analysis

**FIGURE 3:**
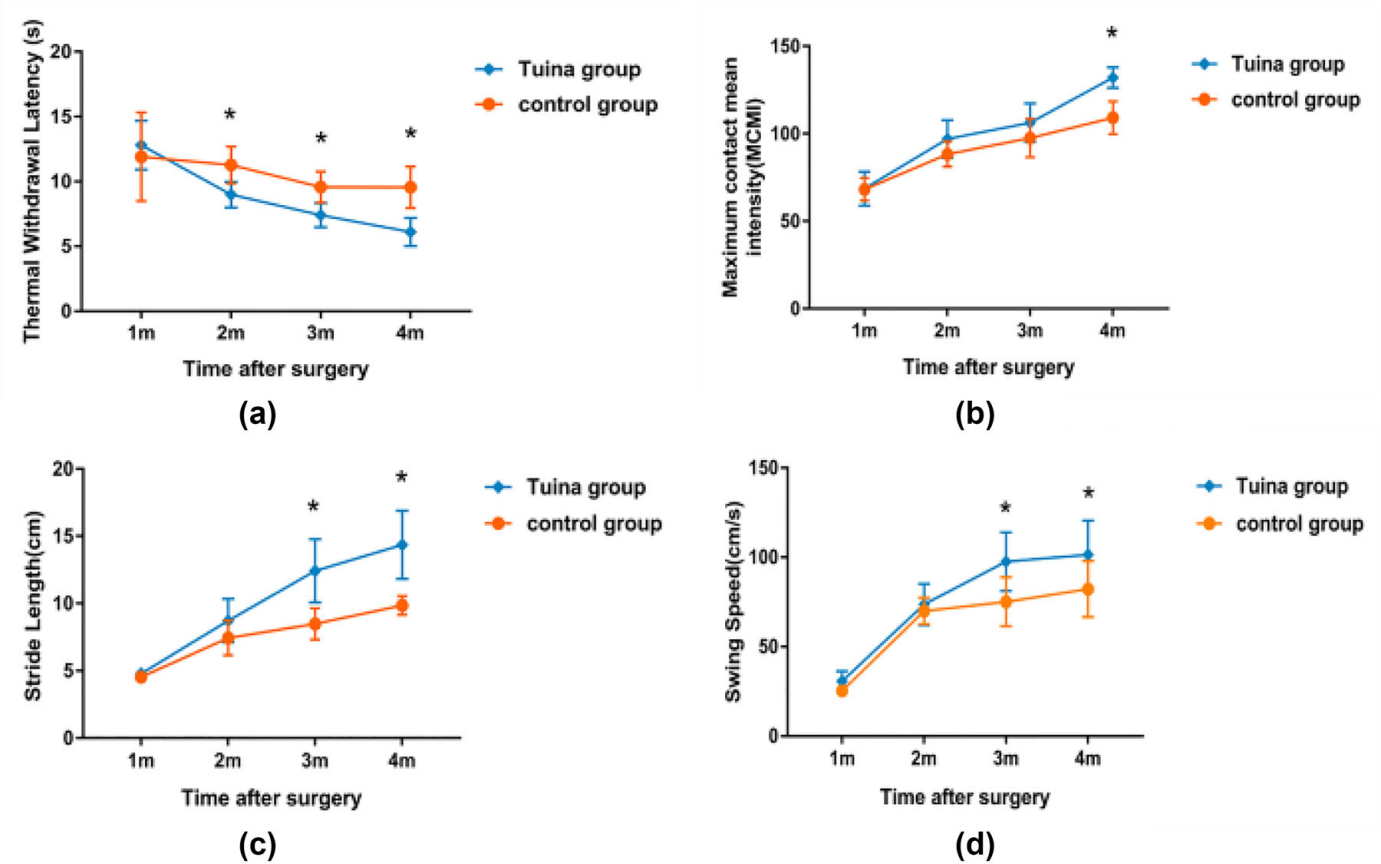
Comparison of behavioral tests between the intervention group and control group. Thermal withdrawal latency (TWL), maximum contact mean intensity (MCMI), stride length(SL), and swing speed (SS) of rats of the intervention group and control group at four time points. *p < .05 (at individual time points between the intervention group and control group).

There was a significant main effect of the group on the TWL, MCMI, SL, and SS ((*F*(1,14) = 12.400, *p* = .003 for the TWL; *F*(1,14) = 21.187, *p* < .001 for the MCMI; *F*(1,14) = 26.060, *p* < .001 for Sthe L; *F*(1,14) = 13.077, *p* = .003 for the SS). Additional two‐sample *t*‐tests were performed between two groups per time point. From the first to fourth postoperative months, the TWL of the two groups both decreased gradually. There was no significant difference between the two groups at first month after surgery (*p* > .05). From the second month, the TWL of the Tuina group was significantly lower than the control group (*p* < .05) (Figure 3a and Table 1). MCMI, SL, and SS of the two groups increased gradually from the first to fourth month. For the MCMI, there was no significant difference between the two groups from the first to third month (*p* > .05). At the fourth month, the MCMI of the Tuina group was significantly higher than the control group (*p* < .05) (Figure 3b and Table 1). For the SL, there was no significant difference between the two groups at the first and the second month (*p* > .05). The SL of the Tuina group was significantly higher than the control group at the third and fourth month (*p* < .05) (Figure 2 and Table 1; Figure 3c and Table 1). For the SS, there was also no significant difference between the two groups at the first and the second month (*p* > .05). At the third and fourth months, the SS of the Tuina group was significantly higher than the control group (*p* < .05) (Figure 3d and Table 1).

**TABLE 1:** Results of behavioral tests and statistical analysis between groups.

Group	Time point	Thermal withdrawal latency (s)	Maximum contact mean intensity (arbitrary unit)	Stride length (cm)	Swing speed (cm/s)
Control group	1 month	11.9 ± 3.42	68.25 ± 6.45	4.55 ± 4.80	26.06 ± 3.21
	2 month	11.27 ± 1.41	88.36 ± 7.20	7.45 ± 1.29	69.90 ± 7.39
	3 month	9.57 ± 1.20	97.54 ± 11.00	8.48 ± 1.16	75.12 ± 13.73
	4 month	9.56 ± 1.60	109.13 ± 9.31	9.86 ± 0.70	82.25 ± 15.64
Tuina group	1 month	12.8 ± 1.88	68.65 ± 9.67	4.80 ± 0.33	30.63 ± 5.70
	2 month	8.99 ± 0.99	97.07 ± 10.72	8.73 ± 1.61	73.65 ± 11.60
	3 month	7.41 ± 0.94	106.29 ± 10.98	12.42 ± 2.35	97.56 ± 16.33
	4 month	6.12 ± 1.08	132.05 ± 5.89	14.36 ± 2.54	101.39 ± 19.14
Control versus Tuina *p*‐value	1 month	.523	.923	.107	.068
	2 month	.002	.077	.101	.454
	3 month	.001	.133	.001	.010
	4 month	.000	.000	.000	.046

### 3.2 fMRI results

The two‐sample *T*‐test was performed between the two groups at each time point. In addition, paired *t*‐tests were conducted to compare the brain activity between the fourth month and the first month within each group. And the threshold of statistical analysis was set at *p* < .05 (FDR correction).

#### 3.2.1 Right (injured) hindpaw stimulation task (intervention group–control group)

**FIGURE 4:**
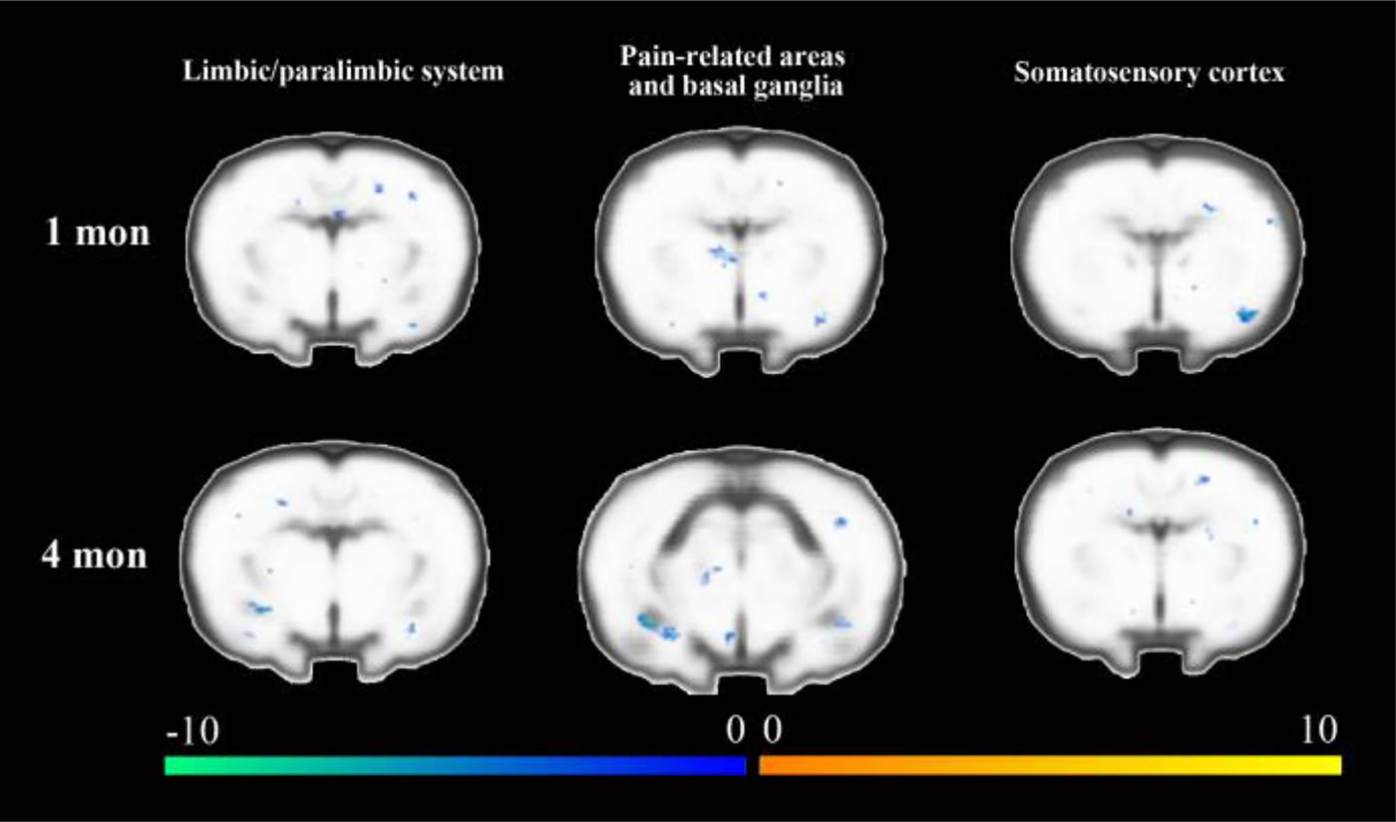
Comparison of cortical activation between the experimental and control groups during the right (injured) hindpaw stimulation task. Each column in the figure displays the difference in the limbic/paralimbic system, pain‐related brain regions, and somatosensory cortex between experimental and control groups. Each row in the figure displays the difference in activation between experimental and control groups at two observation points. The warm tone represents higher activation in the intervention group than that in the control group, while the cold tone represents lower activation.

At the first postoperative month, multiple areas including limbic/paralimbic areas, pain‐regulating areas, and basal ganglia presented lower activation in the intervention group, compared with the control group. Specifically, subregions of the hippocampus, retrosplenial cortex, medial prefrontal cortex, orbitofrontal cortex, hypothalamus, cingulated cortex, piriform cortex, insular, and subregions of thalamus presented significantly lower activation (Figure 4 and Table 2).

**TABLE 2:** Difference of activation between the experimental and control groups during the right (injured) hindpaw stimulation task at the first postoperative month.

		Cluster centroid (MNI)			
Brain regions	Extent	*x*	*y*	*z*	*t*‐value
*Intervention < Control group*					
Cortex_Somatosensory					
(L)cluster1	192	−30	20	−33	−6.323
(L)cluster2	106	−38	15	−43	−7.012
(L)cluster3	15	−63	13	−29	−3.200
Limbic system					
(L)Hippocampus_Subiculum	313	−40	9	27	−15.087
(L)Hippocampus_Postero_Dorsal	313	−34	13	5	−10.148
(L)Cortex_Retrosplenial	244	−3	30	29	−4.037
(L)Hypothalamus_Lateral	61	−1	−32	−1	−5.121
(L)Hippocampus_Antero_Dorsal	46	−3	13	−15	−3.726
(L)Hippocampus_Ventral	41	−50	−20	5	−3.704
(L)Cortex_Medial_Prefrontal	30	−5	5	−83	−4.065
(L)Cortex_Retrosplenial	20	−13	30	9	−3.187
(L)Cortex_Orbitofrontal	17	−40	−7	−79	−2.537
(L)Cortex_Orbitofrontal	10	−3	11	−91	−2.532
(R)Cortex_Retrosplenial	244	16	30	11	−12.421
(R)Hippocampus_Ventral	75	49	−18	7	−3.719
(R)Hippocampus_Ventral	75	57	−20	−5	−4.546
(R)Cortex_Retrosplenial	36	14	30	29	−4.221
(R)Hippocampus_Subiculum	22	22	13	9	−3.446
(R)Hypothalamus_Lateral	21	13	−36	−1	−3.335
(R)Cortex_Piriform	21	36	−18	−79	−3.344
(R)Hippocampus_Postero_Dorsal	16	20	17	−7	−3.710
Pain‐related brain areas					
(L)Cortex_Insular	258	−54	−26	−55	−6.133
(L)Thalamus_Midline_Dorsal	120	−11	−11	−3	−2.446
(L)Thalamus_Dorsolateral	23	−32	−18	1	−4.883
(L)Thalamus_Midline_Dorsal	17	−1	−3	−25	−2.994
(L)Thalamus_Dorsolateral	541	26	−20	−11	−17.900
(L)Thalamus_Midline_Dorsal	118	3	−9	−29	−5.465
(R)Cortex_Cingulate	16	3	17	−37	−5.662

At the fourth postoperative month, the activation pattern was similar to that of the first postoperative month. Areas including the limbic/paralimbic areas, pain‐regulating areas, and basal ganglia presented lower activation in the intervention group. Specific regions of the somatosensory cortex, subregions of the hippocampus, amygdala, AcbC, AcbSh, cingulate cortex, subregions of thalamus, pallidum, and putamen were involved. Additionally, activation in the prefrontal area was higher in the intervention group compared with the control group (Figure 4 and Table 3).

**TABLE 3:** Difference of activation between the experimental and control groups during the right (injured) hindpaw stimulation task at the fourth postoperative month.

		Cluster centroid (MNI)			
Brain regions	Extent	*x*	*y*	*z*	*t*‐value
*Intervention > Control group*					
(L)Cortex_Orbitofrontal	107	−24	−3	−85	3.709
(L)Cortex_Medial_Prefrontal	27	−11	7	−73	2.480
(R)Cortex_Medial_Prefrontal	69	5	3	−83	4.583
(R)Cortex_Frontal_Association	58	30	15	−95	3.649
(R)Cortex_Medial_Prefrontal	37	5	−1	−67	3.159
*Intervention < Control group*					
Cortex_Somatosensory					
(L)cluster1	24	−61	9	−17	−3.158
(L)cluster2	20	−52	11	−23	−3.577
(L)cluster3	10	−50	24	−27	−2.147
(R)cluster4	50	38	22	−53	−3.107
(R)cluster5	39	49	17	−13	−3.926
(R)cluster6	35	42	21	−35	−3.040
(R)cluster7	30	65	7	−51	−2.664
(R)cluster8	17	61	15	−21	−2.683
(R)cluster9	16	34	24	−25	−2.381
Limbic system					
(L)Hippocampus_Subiculum	96	−50	−13	19	−2.250
(L)Cortex_Entorhinal	18	−24	−22	−77	−2.911
(L)Hippocampus_Postero_Dorsal	17	−42	13	−5	−4.283
(L)Hippocampus_Postero_Dorsal	14	−13	7	7	−2.436
(L)Hypothalamus_Medial	13	−11	−42	−21	−2.772
(L)Amygdala	10	−48	−34	−15	−2.859
(L)Amygdala	10	−36	−42	−29	−2.561
(R)Hippocampus_Postero_Dorsal	316	28	21	−7	−4.395
(R)Hypothalamus_Medial	106	7	−38	−21	−3.902
(R)Hippocampus_Antero_Dorsal	48	16	15	−23	−4.160
(R)Amygdala	22	46	−32	−19	−4.215
(R)Hippocampus_Postero_Dorsal	22	53	−5	−3	−3.490
(R)AcbC	21	18	−20	−61	−3.018
(R)Hippocampus_Subiculum	21	24	21	27	−3.374
(R)Hippocampus_Postero_Dorsal	20	3	11	3	−2.710
(R)Hippocampus_Subiculum	12	40	1	25	−2.462
(R)AcbSh	10	7	−30	−59	−2.793
Pain‐related brain areas and basal ganglia					
(L)Cortex_Cingulate	601	−7	24	−41	−6.596
(L)Thalamus_Dorsolateral	82	−24	−11	−1	−2.800
(L)Thalamus_Dorsolateral	49	−24	−3	−31	−2.869
(L)Cortex_Insular	19	−63	−26	−31	−2.971
(L)Thalamus_Midline_Dorsal	19	−1	−14	−9	−2.917
(L)Thalamus_Midline_Dorsal	18	−17	−30	−33	−3.721
(L)Thalamus_Dorsolateral	13	−24	−13	−21	−3.165
(R)Thalamus_Midline_Dorsal	102	16	−9	−5	−3.900
(R)Thalamus_Dorsolateral	48	34	−12	−21	−3.444
(L)Ventral_Pallidum	117	−17	−38	−51	−4.252
(R)Globus_Pallidus	90	36	−30	−27	−3.890
(R)Caudate_Putamen	49	22	−38	−51	−3.399
(R)Caudate_Putamen	37	42	3	−31	−3.283
(L)Caudate_Putamen	18	−13	3	−51	−3.198
(R)Caudate_Putamen	42	34	−14	−41	−2.681

#### 3.2.2 Left (intact)hindpaw stimulation task (intervention group—control group)

**FIGURE 5:**
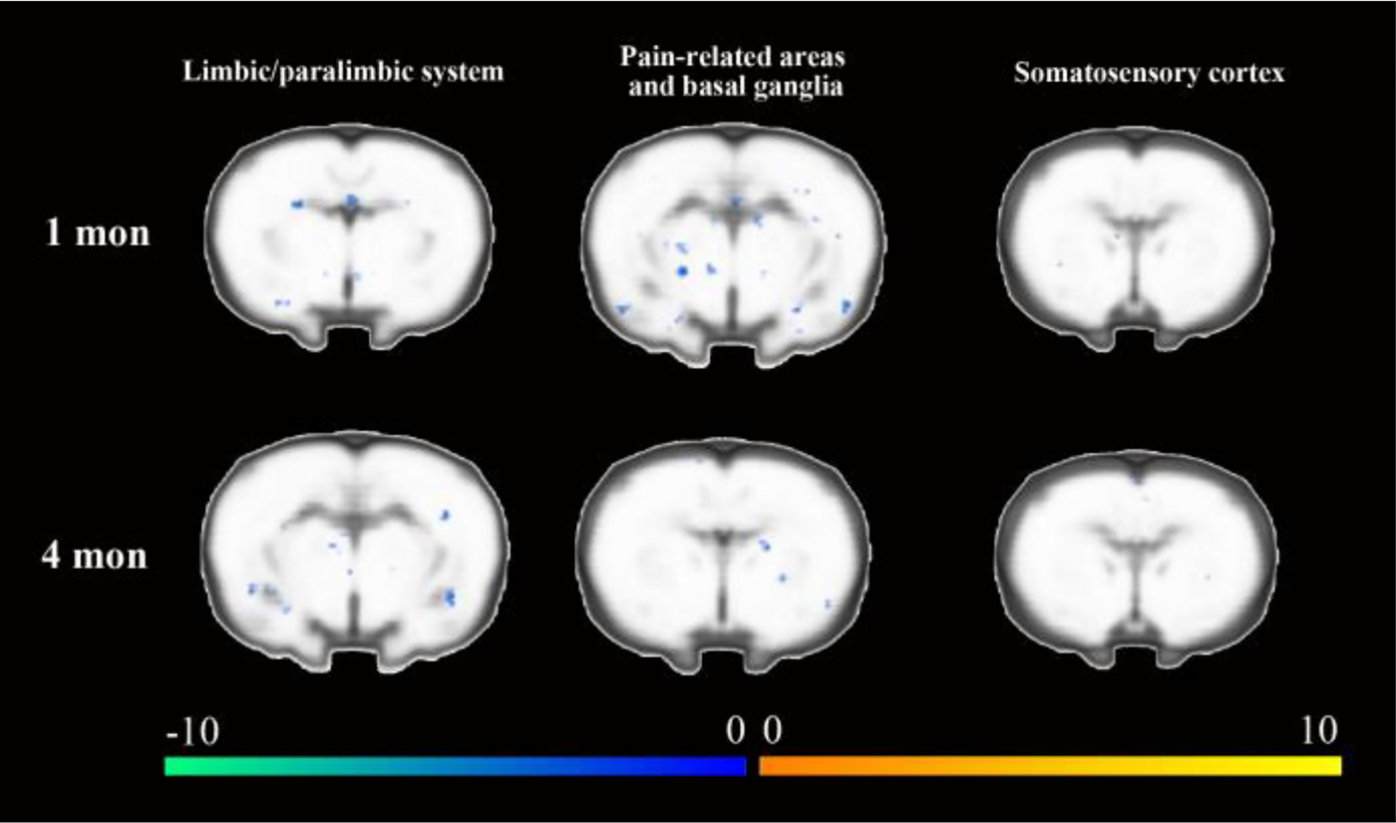
Comparison of cortical activation between the experimental and control groups during the left (intact) hindpaw stimulation task. Each column in the figure displays different activated brain areas between experimental and control groups. Each row in the figure displays the difference in activation between two observation points in the same group. The warm tone represents higher activation in the intervention group than that in the control group, while the cold tone represents lower activation.

At the first postoperative month, activation in multiple areas was lower in the intervention group, including the limbic/paralimbic areas, pain‐regulating areas, and basal ganglia, compared with the control group. Specifically, subregions of the hypothalamus, prefrontal cortex, piriform cortex, entorhinal cortex, cingulate cortex, subregions of the thalamus, pallidum, putamen, and subregions of the hippocampus were involved (Figure 5 and Table 4).

**TABLE 4:** Difference of activation between the experimental and control groups during the left (intact) hindpaw stimulation task at the first postoperative month.

		Cluster centroid (MNI)			
Brain regions	Extent	x	y	z	t‐value
*Intervention < Control group*					
Limbic system					
(L)Hypothalamus_Medial	608	−5	−40	−13	−5.763
(L)Cortex_Entorhinal	22	−59	−34	−27	−4.951
(L)Hippocampus_Antero_Dorsal	15	−30	9	−11	−3.645
(L)Hippocampus_Antero_Dorsal	14	−7	15	−25	−2.923
(L)Hippocampus_Postero_Dorsal	13	−44	3	−3	−4.637
(L)Cortex_Retrosplenial	13	−7	32	−23	−2.276
(R)Hippocampus_Antero_Dorsal	93	1	13	−17	−4.089
(R)Cortex_Medial_Prefrontal	81	7	−1	−67	−2.823
(R)Cortex_Piriform	71	61	−38	−33	−3.100
(R)Hippocampus_Subiculum	52	47	13	15	−3.319
(R)Cortex_Medial_Prefrontal	38	7	−7	−77	−4.016
(R)Hippocampus_Antero_Dorsal	32	30	11	−17	−4.366
(R)Hypothalamus_Medial	12	3	−38	−35	−3.328
(R)Hippocampus_Subiculum	11	34	9	27	−3.489
Pain‐related brain areas and basal ganglia					
(L)Cortex_Cingulate	81	−3	15	−61	−4.181
(L)Thalamus_Ventromedial	35	−3	−28	−33	−5.730
(L)Cortex_Insular	15	−59	−15	−55	−3.184
(R)Thalamus_Dorsolateral	69	14	−20	−19	−3.481
(R)Thalamus_Dorsolateral	29	30	−20	13	−5.650
(R)Ventral_Pallidum	28	24	−30	−45	−6.986
(R)Substantia_Nigra	23	18	−34	9	−4.735
(R)Caudate_Putamen	784	44	−14	−47	−3.346
(R)Ventral_Pallidum	6	34	−40	−43	−2.739
(R)Cortex_Medial_Prefrontal	81	7	−1	−67	−2.823
(R)Cortex_Medial_Prefrontal	38	7	−7	−77	−4.016
(R)Cortex_Parietal_Association	187	42	23	−1	−2.681

At the fourth postoperative month, activation in brain regions in the intervention group was also lower, involving the limbic/paralimbic areas, pain‐regulating areas, and basal ganglia, compared with the control group. Specifically, the retrosplenial cortex, AcbSh, subregions of the hypothalamus, insular cortex, globus pallidus, cingulate cortex, subregions of thalamus, pallidum, and putamen were involved (Figure 5 and Table 5).

**TABLE 5:** Difference of activation between the experimental and control groups during the left (intact) hindpaw stimulation task at the fourth postoperative month.

		Cluster centroid (MNI)			
Brain regions	Extent	*x*	*y*	*z*	*t*‐value
*Intervention < Control group*					
Limbic system					
(L)Cortex_Retrosplenial	30	−1	38	−19	−2.766
(L)Cortex_Retrosplenial	20	−19	24	27	−3.919
(L)AcbSh	20	−15	−32	−67	−3.309
(L)Cortex_Retrosplenial	13	−17	28	11	−3.391
(L)Hypothalamus_Medial	13	−3	−26	−7	−2.294
(L)Cortex_Retrosplenial	10	−3	22	−21	−2.497
(R)Hippocampus_Ventral	37	61	−18	1	−5.351
(R)Hippocampus_Antero_Dorsal	36	12	19	−15	−3.697
(R)Hippocampus_Postero_Dorsal	36	26	26	−7	−3.405
(R)Hypothalamus_Medial	17	3	−44	−9	−2.648
Pain‐related brain areas and basal ganglia					
(L)Thalamus_Dorsolateral	26	−19	−18	−11	−4.064
(L)Cortex_Insular	23	−57	−26	−45	−3.107
(L)Thalamus_Midline_Dorsal	20	−7	−3	−23	−2.374
(L)Cortex_Insular	10	−61	−9	−57	‐2.893
(R)Thalamus_Midline_Dorsal	39	9	−7	−17	−2.761
(R)Thalamus_Dorsolateral	25	38	3	−5	−2.603
(R)Thalamus_Dorsolateral	14	32	−16	−13	−2.574
(L)Globus_Pallidus	139	−32	−13	−39	−3.514
(L)Caudate_Putamen	43	−15	−1	−49	−2.565
(R)Caudate_Putamen	15	16	−13	−49	−3.006
(R)Caudate_Putamen	13	16	7	−43	−2.391
(R)Caudate_Putamen	55	38	−11	−57	−4.088
(L)Cortex_Frontal_Association	76	−34	16	−95	−2.828

#### 3.2.3 Right (injured) hindpaw stimulation task (the first postoperative month—the fourth postoperative month)

In the control group, compared to the first month, the following brain regions showed increased activity in the fourth month: right posterodorsal hippocampus, the right interstitial nucleus of the posterior limb of the anterior commissure (IPAC); The following brain regions showed decreased activity: left retrosplenial cortex, right entorhinal cortex, left superior colliculus, left motor cortex, right mesencephalic region (Table 6).

**TABLE 6:** Difference of activation between the fourth month and the first month during the right (injured) hindpaw stimulation task in the brain regions.

		Cluster centroid (MNI)			
Brain regions	Extent	*x*	*y*	*z*	*t*‐value
Control group					
*Fourth month > First month*					
(R)Hippocampus_Postero_Dorsal	51	51	1	−3	23
(R)IPAC	63	28	−42	−47	63
*Fourth month < First month*					
L_Cortex_Retrosplenial	56	−3	26	−9	−44
R_Cortex_Entorhinal	69	53	−30	−39	−42
L_Superior_Colliculus	47	−24	−7	19	−32
L_Cortex_Motor	51	−13	30	−49	−20
R_Mesencephalic_Region	39	7	−26	23	−19
Intervention					
*Fourth month < First month*					
Frontal_Sup_2_R	20	34	−10	71	−16

In the intervention group, the following brain region showed decreased activity in the fourth month compared to the first month: right superior frontal (Table 6).

#### 3.2.4 Motor‐related brain regions during right (injured) hindpaw stimulation task (intervention group–control group)

At the first postoperative month, the control group showed increased activity in the primary motor cortex and somatosensory cortex of the right hemisphere compared to the intervention group (Table 7), *p* < .001.

**TABLE 7:** Difference of activation between the experimental and control groups during right (injured) hindpaw stimulation task in the motor‐related brain regions.

		Cluster centroid (MNI)			
Brain regions	Extent	*x*	*y*	*z*	*t*‐value
First month					
*Intervention < Control group*					
(R)Cortex_Motor	12	12	34	−37	−2.524
(R)Cortex_Somatosensory	69	59	−16	−3	−4.029
Fourth month					
*Intervention > Control group*					
(L)Cortex_Motor	15	−32	24	−89	2.598
(R)Cortex_Motor	24	30	20	−83	2.944
(R)Cortex_Somatosensory	26	61	−16	7	2.818

At the fourth postoperative month, the intervention group showed increased activity in both primary motor cortices and the somatosensory cortex of the right hemisphere compared to the control group (Table 7), *p* < .001.

### 3.3 Correlation

At the first postoperative month, the Pearson correlation coefficients between the TWL of the injured hindpaw and the functional activity of the left insular cortex was –.03210 (*p* > .05), indicating no statistical significance; the Pearson correlation coefficients between the right motor cortex and the MCMI, SL, and SS of the injured limb were .1602, −.01238, −.01712 (*p* > .05), indicating no statistical significance.

At the fourth postoperative month, the Pearson correlation coefficients between the right motor cortex and the MCMI, SL, and SS of the injured limb were .5326, −.1351, −.2623 (*p* > .05), indicating no statistical significance; the Pearson correlation coefficients between the left motor cortex and the MCMI, SL, and SS of the injured limb were .2446, .2945, −.02689 (*p* > .05), indicating no statistical significance.

## 4 DISCUSSION

The sciatic nerve is a mixed nerve that composes of sensory and motor fibers. After injury, both motor and sensory functions of the hindlimb are partially or completely lost except for the saphenous innervations area (Shin & Howard, 2012).

Several measures could be used to evaluate sensorimotor function recovery following sciatic nerve injury repair. The TWL is one of the classic methods to evaluate the recovery of sensory function after sciatic nerve injury in rats. It can quantitatively evaluate the sensory function recovery of injured hindlimbs (da Silva et al., 2022). In the present study, the sensory function gradually recovered in both the experimental and control groups, indicated by decreased TWL. Tuina could significantly promote the final outcome of the sensory function following nerve repair.

Gait analysis is an automated behavior testing method, which is easy to control the speed of animal movement and allows the evaluation of animal locomotion function in free status (Bozkurt et al., 2008; Xu et al., 2019) and has been widely used in animal models of peripheral nervous diseases. It is also an important method for evaluating static and dynamic functions after nerve injury (Deumens et al., 2007; Gabriel et al., 2007; Kappos et al., 2017; Wu, Lu, Hua, Ma, Shan, et al., 2018; Wu, Lu, Hua, Ma, & Xu, 2018). In the present study, gait analysis parameters, MCMI, SL, and SS, were used to evaluate the motor function after sciatic nerve injury repair. The motor function recovered to various degrees in both groups. The MCMI, SL, and SS were significantly better in the Tuina group. Therefore, Tuina is also related to better motor recovery after nerve repair. The effect was more obvious at the third and fourth months after the surgery.

Previous studies have demonstrated that peripheral nerve injuries would result in changes not only at the local site of injury but also in long‐lasting cortical plasticity. These changes are caused by permanent loss of sensation and the misdirection of the newly formed axons after the nerve repair, the newly formed sensory and motor axons reinnervated do not match their original organs (Darian‐Smith, 1994; Lundborg, 2007; Rosen & Lundborg, 2004; Yamahachi et al., 2009). Reports have suggested that Tuina would improve functional outcomes following peripheral nerve injury repair (Z. Liu et al., 2021; T. T. Lv, Mo, Yu, Shao, et al., 2020). However, it is not well understood whether the long‐lasting cortical plasticity is involved in the improvement of function after Tuina therapy. To our knowledge, the present study is the first longitudinal study on cortical plasticity in a rat model of peripheral nerve injury treated by Tuina therapy.

Our study found that 1 month after modeling, compared to the intervention group, the primary motor cortex activity in the right hemisphere was enhanced in the control group, possibly due to the compensatory movement of the contralateral limb caused by pain in the affected limb of the control group, which promoted the activation of the right motor cortex. Interestingly, after 4 months of modeling, the activity of the bilateral motor cortex was higher in the intervention group than in the control group. While the longitudinal comparison of the model group showed a decrease in activity in the left primary motor cortex, there was no significant change in the intervention group. Behavioral results also indicated that the intervention group outperformed the control group, which may reflect the preservation effect of Tuina on the limb motor function after peripheral nerve injury, avoiding a decline in the motor function caused by nerve damage.

In the cortical activation regions during right (injured) hindpaw stimulation tasks, several brain areas were less activated in the intervention group than that in the control group. As the somatosensory cortex mainly receives a projection of sensory fibers from the peripheral, and the enhancement of afferent signal is a key factor that impacts the excitability of the somatosensory cortex. A study on monkeys has proved this view. They found that high‐intensity stimulation to the monkey`s fingers can make the somatosensory cortex more activated (Qi et al., 2016). It was also reported that Tuina therapy may decrease the activation level of peripheral nociceptive C‐fiber, which might reduce the sensory afferentation signal (Jiang et al., 2016). Therefore, the somatosensory cortex of the intervention group showed lower activation during the peripheral stimulating task.

Neuropathies following transection usually induce paresthesia or pain before reinnervation of targets (Alexander et al., 2019). Tuina therapy would alleviate paresthesia or pain after the peripheral neural pathway was reconnected. This reduction of sensory afferents following Tuina therapy consequently led to a reduction in the extent and strength of activation in the sensory cortex. Therefore, the somatosensory cortex in the intervention group was less activated.

In addition, the pain‐related areas, basal ganglia, and limbic/paralimbic areas also presented lower activation in the Tuina group. Previous studies have reported regions of modules in the “pain network,” including the primary somatosensory cortex, medial frontal cortical structures (such as the ACC, middle cingulate cortex (MCC), and supplementary motor area (SMA)), and the parasylvian cortical structures (such as the insula and opercular) (C. C. Liu et al., 2011). The insula is most strongly associated with other structures in the brain network, the anterior insula is connected with ACC and the frontal cortices, while the posterior insula is associated with the primary somatosensory cortex, motor cortex, and temporal cortex (Abram et al., 2015). The ACC is a key structure that processes information relating to pain‐induced unpleasantness. The thalamus is an important sensory relay station that transmits sensory signals to the cerebral cortex and is highly associated with pain (Zitnik et al., 2014). The basal ganglia (such as caudate, putamen, and pallidum) is a major site for adaptive plasticity in the brain, affecting the normal state in a broad range of behaviors as well as neurological and psychiatric conditions including pain (Graybiel, 2004). The limbic system includes multiple brain regions involving memory, emotion, and sensation. The hippocampus and amygdala transmit cortical signals to the hypothalamus and brainstem to regulate the arousal and motivational state of the whole neuraxis (Cardinal, 2002). It is recognized that the hippocampus, amygdala, and parahippocampus play an important role in regulating motivation, emotions, memory, and pain (Kirby et al., 2013; Y. Yang et al., 2016; Zubieta et al., 2001). Moreover, the inactivation of the hippocampus and amygdala was found to be related to the regulation of pain threshold (W. T. Zhang et al., 2003). In the present study, the activation pattern of pain‐related areas, basal ganglia, and limbic/paralimbic areas was quite synchronized. Therefore, these areas also played an important role in Tuina therapy‐induced brain plasticity.

The prefrontal cortex has been suggested to participate in controlling functional interactions between areas of the pain‐related brain regions (Tracey & Mantyh, 2007). Along with the basal ganglia, the prefrontal cortex is also closely associated with attention and executive during tasks (Yuan & Raz, 2014). Considering the integration of sensory and motor functions, the sensory input is essential for motor initialization and adjustment in walking. After 4 months of Tuina therapy, the higher activation of the prefrontal cortex implied restoration of the motor regulating function. Rather than reactivating some isolated areas alone, it demonstrated a gradual recovery of more advanced brain function.

According to the brain activation regions during the left (intact) hindpaw stimulation task, lower activation of pain‐related areas, basal ganglia, and limbic/paralimbic was observed in the intervention group, which was similar to the right (injured)hindpaw stimulation task. We assumed that lower activation in pain‐related areas and advanced modulation network was related to relieving effects of Tuina therapy on decreasing the excitability of sensory, pain, and emotion‐related areas. This suggested that Tuina therapy potentially leads to more extended changes in the brain other than the local effect.

## 5 CONCLUSION

Tuina therapy on the gastrocnemius muscle of rats with sciatic nerve injury can effectively alleviate pain and maintain the motor function of the affected limb, thus avoiding the decline in activity caused by nerve injury. In addition, Tuina therapy changes the activation level of pain‐related brain regions and the decreased activity of the motor cortex caused by nerve injury, providing a central nervous system mechanism for Tuina therapy in peripheral nerve injury and reflecting the impact of peripheral stimulation on brain plasticity.

### 5.1 Limitation

Further investigations should be conducted before extrapolating the present conclusions due to the differences between humans and rodents. More investment is needed to study the central nervous system mechanisms of Tuina therapy.

## AUTHOR CONTRIBUTIONS

S‐JM, J‐JW: conceptualization and writing–original draft. X‐YH, M‐XZ: methodology. S‐JM, J‐PZ, and X‐YH: validation. J‐JW and M‐XZ: formal analysis. J‐GX and J‐PZ: writing, reviewing, and editing. All authors read and approved the final manuscript.

## CONFLICT OF INTEREST STATEMENT

The authors declare that the research was conducted in the absence of any commercial or financial relationships that could be construed as a potential conflict of interest.

### PEER REVIEW

The peer review history for this article is available at https://publons.com/publon/10.1002/brb3.3174.

## Data Availability

The raw data supporting the conclusions of this article will be made available by the authors, without undue reservation.

## References

[brb33174-bib-0001] Abram, S. V. , Wisner, K. M. , Grazioplene, R. G. , Krueger, R. F. , Macdonald, A. W. , & Deyoung, C. G. (2015). Functional coherence of insula networks is associated with externalizing behavior. Journal of Abnormal Psychology, 124(4), 1079–1091. 10.1037/abn0000078 26301974 PMC4658249

[brb33174-bib-0002] Alexander, J. H. , Jordan, S. W. , West, J. M. , Compston, A. , Fugitt, J. , Bowen, J. B , Dumanian, G. A. , Pollock, R. , Mayerson, J. L. , Scharschmidt, T. J. , & Valerio, I. L. (2019). Targeted muscle reinnervation in oncologic amputees: Early experience of a novel institutional protocol. Journal of Surgical Oncology, 120(3), 348–358. 10.1002/jso.25586 31197851 PMC7701996

[brb33174-bib-0003] Bao, B.‐Bo. , Zhu, H.‐Yi. , Wei, H.‐F. , Li, J. , Wang, Z.‐B. , Li, Y.‐H. , Hua, Xu.‐Y. , Zheng, M.‐X. , & Zheng, X.‐Y. (2022). Altered intra‐ and inter‐network brain functional connectivity in upper‐limb amputees revealed through independent component analysis. Neural Regeneration Research, 17(12), 2725–2729. 10.4103/1673-5374.339496 35662220 PMC9165370

[brb33174-bib-0004] Bao, Q. , Liu, Q. , Wang, J. , Shen, Y. , & Zhang, W. (2021). Impaired limb functional outcome of peripheral nerve regeneration is marked by incomplete recovery of paw muscle atrophy and brain functional connectivity in a rat forearm nerve repair model. Neural Plasticity, 2021, 6689476. 10.1155/2021/6689476 33628221 PMC7892249

[brb33174-bib-0005] Bhat, D. I. , Indira Devi, B. , Bharti, K. , & Panda, R. (2017). Cortical plasticity after brachial plexus injury and repair: A resting‐state functional MRI study. Neurosurgical Focus [Electronic Resource], 42(3), E14. 10.3171/2016.12.FOCUS16430 28245732

[brb33174-bib-0006] Bozkurt, A. , Deumens, R. , Scheffel, J. , O'dey, D. M. , Weis, J. , Joosten, E. A. , Führmann, T. , Brook, G. A. , & Pallua, N. (2008). CatWalk gait analysis in assessment of functional recovery after sciatic nerve injury. Journal of Neuroscience Methods, 173(1), 91–98. 10.1016/j.jneumeth.2008.05.020 18577402

[brb33174-bib-0007] Cabanas‐Valdés, R. , Calvo‐Sanz, J. , Serra‐Llobet, P. , Alcoba‐Kait, J. , González‐Rueda, V. , & Rodríguez‐Rubio, P. R. (2021). The Effectiveness of massage therapy for improving sequelae in post‐stroke survivors. A systematic review and meta‐analysis. International Journal of Environmental Research and Public Health, 18(9), 4424. 10.3390/ijerph18094424 33919371 PMC8122530

[brb33174-bib-0008] Cardinal, R. N. , Parkinson, J. A. , Hall, J. , & Everitt, B. J. (2002). Emotion and motivation: The role of the amygdala, ventral striatum, and prefrontal cortex. Neuroscience and Biobehavioral Reviews, 26, 321–352. 10.1016/s0149-7634(02)00007-6 12034134

[brb33174-bib-0009] Darian‐Smith, C. , & Gilbert, C. D. (1994). Axonal sprouting accompanies functional reorganization in adult cat striate cortex. Nature, 368, 737–740. 10.1038/368737a0 8152484

[brb33174-bib-0010] Da Silva, F. B. O. , Santos, M. D. C. Q , Da Silva, T. C. B. , Facchini, D. , Kolberg, A. , Barros, R. R. , Silveira, E. M. S. , Kroth, A. , Duarte, F. C. K. , Vassoler, J. M. , Kolberg, C. , & Partata, W. A. (2022). Spine‐adjusting instrument (Impulse(R)) attenuates nociception and modulates oxidative stress markers in the spinal cord and sciatic nerve of a rat model of neuropathic pain. Pain Medicine (Malden, Mass.), 23(4), 761–773. 10.1093/pm/pnab167 33993301

[brb33174-bib-0011] Deumens, R. , Jaken, R. J. P. , Marcus, M. A. E. , & Joosten, E. A. J. (2007). The CatWalk gait analysis in assessment of both dynamic and static gait changes after adult rat sciatic nerve resection. Journal of Neuroscience Methods, 164(1), 120–130. 10.1016/j.jneumeth.2007.04.009 17532474

[brb33174-bib-0012] Fang, L. , Fang, M. , & Guo, M.‐M. (2018). Study on force mechanism for therapeutic effect of pushing manipulation with one‐finger meditation base on similarity analysis of force and waveform. Chinese Journal of Integrative Medicine, 24(7), 531–536. 10.1007/s11655-016-2275-x 28028714

[brb33174-bib-0013] Fox, M. D. (2018). Mapping symptoms to brain networks with the human connectome. The New England Journal of Medicine, 379(23), 2237–2245. 10.1056/NEJMra1706158 30575457

[brb33174-bib-0014] Gabriel, A. F. , Marcus, M. A. E. , Honig, W. M. M. , Walenkamp, G. H. I. M. , & Joosten, E. A. J. (2007). The CatWalk method: A detailed analysis of behavioral changes after acute inflammatory pain in the rat. Journal of Neuroscience Methods, 163(1), 9–16. 10.1016/j.jneumeth.2007.02.003 17382401

[brb33174-bib-0015] Graybiel, A. M. (2004). Network‐level neuroplasticity in cortico‐basal ganglia pathways. Parkinsonism & Related Disorders, 10(5), 293–296. 10.1016/j.parkreldis.2004.03.007 15196508

[brb33174-bib-0016] Guo, X. , Yu, T.‐Y. , Steven, W. , Jia, W.‐D. , Ma, C. , Tao, Y.‐H. , Yang, C. , Lv, T.‐T. , Wu, S. , Lu, M.‐Q. , & Liu, J.‐Li. (2016). Three methods and three points regulates p38 mitogen‐activated protein kinase in the dorsal horn of the spinal cord in a rat model of sciatic nerve injury. Neural Regeneration Research, 11(12), 2018–2024. 10.4103/1673-5374.197147 28197201 PMC5270443

[brb33174-bib-0017] Happe, S. , Peikert, A. , Siegert, R. , & Evers, S. (2016). The efficacy of lymphatic drainage and traditional massage in the prophylaxis of migraine: A randomized, controlled parallel group study. Neurological Sciences, 37(10), 1627–1632. 10.1007/s10072-016-2645-3 27338942

[brb33174-bib-0018] He, Q.‐R. , Cong, M. , Yu, F.‐H. , Ji, Y.‐H. , Yu, S. , Shi, H.‐Y. , & Ding, F. (2022). Peripheral nerve fibroblasts secrete neurotrophic factors to promote axon growth of motoneurons. Neural Regeneration Research, 17(8), 1833–1840. 10.4103/1673-5374.332159 35017446 PMC8820717

[brb33174-bib-0019] Huang, F. , Zhao, S. , Dai, L. , Feng, Z. , Wu, Z. , Chen, J. , Guo, R. , Tian, Q. , Fan, Z. , & Wu, S. (2020). Tuina for cervical vertigo: A systematic review and meta‐analysis of randomized controlled trials. Complementary Therapies in Clinical Practice, 39, 101115. 10.1016/j.ctcp.2020.101115 32379654

[brb33174-bib-0020] Huang, S.‐R. (2003). Pathogenic factors of blood circulation disturbance in lumbar intervertebral disc herniation and mechanism of Tuina manipulation in promoting circulation. Zhong Xi Yi Jie He Xue Bao[ Journal of Chinese Integrative Medicine], 1, 255–258. 10.3736/jcim20030406 15339523

[brb33174-bib-0021] Jenkins, L. C. , Chang, W.‐J. , Buscemi, V. , Liston, M. , Humburg, P. , Nicholas, M. , Graven‐Nielsen, T. , Hodges, P. W. , Mcauley, J. H. , & Schabrun, S. M. (2023). Cortical function and sensorimotor plasticity are prognostic factors associated with future low back pain after an acute episode: The understanding persistent Pain Where it ResiDes Prospective Cohort Study. Pain, 164(1), 14–26. 10.1097/j.pain.0000000000002684 35559930

[brb33174-bib-0022] Jiang, S. , Zhang, H. , Fang, M. , Zhang, Y. , Lu, N. , Zhu, Q. , Cheng, Y. , Ai, J. , Zhou, N. , Li, J. , Fang, L. , & Yao, F. (2016). Analgesic effects of Chinese Tuina massage in a rat model of pain. Experimental and Therapeutic Medicine, 11(4), 1367–1374. 10.3892/etm.2016.3055 27073451 PMC4812208

[brb33174-bib-0023] Kang, Z. , Xing, H. , Lin, Q. , Meng, F. , & Gong, Li. (2022). Effectiveness of therapeutic massage for improving motor symptoms in Parkinson's disease: A systematic review and meta‐analysis. Frontiers in Neurology, 13, 915232. 10.3389/fneur.2022.915232 36133798 PMC9483027

[brb33174-bib-0024] Kappos, E. A. , Sieber, P. K. , Engels, P. E. , Mariolo, A. V. , D'arpa, S. , Schaefer, D. J. , & Kalbermatten, D. F. (2017). Validity and reliability of the CatWalk system as a static and dynamic gait analysis tool for the assessment of functional nerve recovery in small animal models. Brain and Behavior, 7(7), e00723. 10.1002/brb3.723 28729931 PMC5516599

[brb33174-bib-0025] Kirby, E. D. , Muroy, S. E. , Sun, W. G. , Covarrubias, D. , Leong, M. J. , Barchas, L. A. , & Kaufer, D. (2013). Acute stress enhances adult rat hippocampal neurogenesis and activation of newborn neurons via secreted astrocytic FGF2. Elife, 2, e00362. 10.7554/eLife.00362 23599891 PMC3628086

[brb33174-bib-0026] Li, H. , Li, X. , Wang, J. , Gao, F. , Wiech, K. , Hu, Li. , & Kong, Y. (2022). Pain‐related reorganization in the primary somatosensory cortex of patients with postherpetic neuralgia. Human Brain Mapping, 43(17), 5167–5179. 10.1002/hbm.25992 35751551 PMC9812237

[brb33174-bib-0027] Li, R. , Liu, Z. , Pan, Y. , Chen, L. , Zhang, Z. , & Lu, L. (2014). Peripheral nerve injuries treatment: A systematic review. Cell Biochemistry and Biophysics, 68(3), 449–454. 10.1007/s12013-013-9742-1 24037713

[brb33174-bib-0028] Li, T. , Li, X. , Huang, F. , Tian, Q. , Fan, Z. Y. , & Wu, S. (2021). Clinical efficacy and safety of acupressure on low back pain: A systematic review and meta‐analysis. Evidence‐Based Complementary and Alternative Medicine, 2021, 8862399. 10.1155/2021/8862399 33708260 PMC7932783

[brb33174-bib-0029] Liu, C. C. , Franaszczuk, P. , Crone, N. E. , Jouny, C. , & Lenz, F. A. (2011). Studies of properties of “Pain Networks” as predictors of targets of stimulation for treatment of pain. Front Integr Neurosci, 5, 80–86. 10.3389/fnint.2011.00080 22164137 PMC3230069

[brb33174-bib-0030] Liu, Z. , Wang, H. , Yu, T. , Jiao, Yi. , Zhang, Y. , Liu, Di. , Xu, Y. , Guan, Q. , & Lu, M. (2021). A Review on the Mechanism of Tuina Promoting the Recovery of Peripheral Nerve Injury. Evidence‐Based Complementary and Alternative Medicine, 2021, 6652099. 10.1155/2021/6652099 34285705 PMC8275372

[brb33174-bib-0031] Liu, Z.‐F. , Wang, H.‐R. , Yu, T.‐Y. , Zhang, Y.‐Q. , Jiao, Y. , & Wang, X.‐Y. (2022). Tuina for peripherally‐induced neuropathic pain: A review of analgesic mechanism. Front Neurosci, 16, 1096734. 10.3389/fnins.2022.1096734 36620462 PMC9817144

[brb33174-bib-0032] Lundborg, G. , Björkman, A. , & Rosén, B. (2007). <Enhanced sensory relearning after nerve repair by using repeated forearm anaesthesia aspects on time dynamics of treatment.pdf>. Acta Neurochirurgica. Supplement, 100, 121–126. 10.1007/978-3-211-72958-8_26 17985560

[brb33174-bib-0033] Lv, T. , Mo, Y. , Yu, T. , Zhang, Y. , Shao, S. , Luo, Y. , Shen, Yi. , Lu, M. , & Wong, S. G. (2020). An Investigation into the Rehabilitative Mechanism of Tuina in the Treatment of Sciatic Nerve Injury. Evidence‐Based Complementary and Alternative Medicine, 2020, 5859298. 10.1155/2020/5859298 32724326 PMC7382723

[brb33174-bib-0034] Lv, T.‐T. , Mo, Y.‐J. , Yu, T.‐Y. , Shao, S. , Lu, M.‐Q. , Luo, Y.‐T. , Shen, Y. , Zhang, Y.‐M. , & Steven, W. (2020). Using RNA‐Seq to explore the repair mechanism of the three methods and three‐acupoint technique on DRGs in Sciatic nerve injured rats. Pain Research & Management, 2020, 7531409. 10.1155/2020/7531409 32587647 PMC7298348

[brb33174-bib-0035] Navarro, X. , Vivó, M. , & Valero‐Cabré, A. (2007). Neural plasticity after peripheral nerve injury and regeneration. Progress in Neurobiology, 82(4), 163–201. 10.1016/j.pneurobio.2007.06.005 17643733

[brb33174-bib-0036] Nie, L. , Cheng, J. , Wen, Y. , & Li, J. (2019). The Effectiveness of acupuncture combined with Tuina therapy in patients with migraine. Complementary Medicine Research, 26(3), 182–194. 10.1159/000496032 30893677

[brb33174-bib-0037] Pach, D. , Piper, M. , Lotz, F. , Reinhold, T. , Dombrowski, M. , Chang, Y. , Liu, B. , Blödt, S. , Rotter, G. , Icke, K. , & Witt, C. M. (2018). Effectiveness and cost‐effectiveness of Tuina for Chronic neck pain: A randomized controlled trial comparing Tuina with a no‐intervention waiting list. Journal of Alternative and Complementary Medicine, 24(3), 231–237. 10.1089/acm.2017.0209 29072931

[brb33174-bib-0038] Pallarés, V. , Moya, J. , Samper‐Belda, F. J. , Canals, S. , & Moratal, D. (2015). Neurosurgery planning in rodents using a magnetic resonance imaging assisted framework to target experimentally defined networks. Computer Methods and Programs in Biomedicine, 121(2), 66–76. 10.1016/j.cmpb.2015.05.011 26094858

[brb33174-bib-0039] Qi, H.‐X. , Wang, F. , Liao, C.‐C. , Friedman, R. M. , Tang, C. , Kaas, J. H. , & Avison, M. J. (2016). Spatiotemporal trajectories of reactivation of somatosensory cortex by direct and secondary pathways after dorsal column lesions in squirrel monkeys. Neuroimage, 142, 431–453. 10.1016/j.neuroimage.2016.08.015 27523450 PMC5159255

[brb33174-bib-0040] Ren, W. , Centeno, M. V. , Berger, S. , Wu, Y. , Na, X. , Liu, X. , Kondapalli, J. , Apkarian, A. V. , Martina, M. , & Surmeier, D. J. (2016). The indirect pathway of the nucleus accumbens shell amplifies neuropathic pain. Nature Neuroscience, 19(2), 220–222. 10.1038/nn.4199 26691834 PMC4889808

[brb33174-bib-0041] Rocca, M. A. , & Filippi, M. (2006). Functional MRI to study brain plasticity in clinical neurology. Neurological Sciences, 27(Suppl 1), s24–s26. 10.1007/s10072-006-0541-y 16708177

[brb33174-bib-0042] Rosen, B. , & Lundborg, G. (2004). Sensory re‐education after nerve repair: Aspects of timing. Handchirurgie, Mikrochirurgie, Plastische Chirurgie, 36(1), 8–12. 10.1055/s-2004-815808 15083384

[brb33174-bib-0043] Ruven, C. , Li, W. , Li, H. , Wong, W.‐M. , & Wu, W. (2017). Transplantation of embryonic spinal cord derived cells helps to prevent muscle atrophy after peripheral nerve injury. International Journal of Molecular Sciences, 18(3), 511. 10.3390/ijms18030511 28264437 PMC5372527

[brb33174-bib-0044] Shan, A. , Zhang, H. , Gao, M. , Wang, L. , Cao, X. , Gan, C. , Sun, H. , Yuan, Y. , & Zhang, K. (2023). Aberrant voxel‐based degree centrality and functional connectivity in Parkinson's disease patients with fatigue. CNS Neuroscience & Therapeutics, Advance online publication. 10.1111/cns.14212 PMC10401083 37032641

[brb33174-bib-0045] Shin, J. H. , & Howard, F. M. (2012). Abdominal wall nerve injury during laparoscopic gynecologic surgery: Incidence, risk factors, and treatment outcomes. Journal of Minimally Invasive Gynecology, 19(4), 448–453. 10.1016/j.jmig.2012.03.009 22560041

[brb33174-bib-0046] Sousa, C. M. , Coimbra, D. , Machado, J. , & Greten, H. J. (2015). Effects of self‐administered exercises based on Tuina techniques on musculoskeletal disorders of professional orchestra musicians: A randomized controlled trial. Journal of Integrative Medicine, 13(5), 314–318. 10.1016/S2095-4964(15)60194-7 26343102

[brb33174-bib-0047] Sousa, C. M. , Moreira, L. , Coimbra, D. , Machado, J. , & Greten, H. J. (2015). Immediate effects of Tuina techniques on working‐related musculoskeletal disorder of professional orchestra musicians. Journal of Integrative Medicine, 13(4), 257–261. 10.1016/S2095-4964(15)60181-9 26165370

[brb33174-bib-0048] Sun, J.‐H. , Huang, M. , Fang, Z. , Li, T.‐X. , Wu, T.‐T. , Chen, Y. , Quan, D.‐P. , Xu, Y.‐Y. , Wang, Y.‐M. , Yang, Y. , & Zou, J.‐L. (2022). Nerve bundle formation during the promotion of peripheral nerve regeneration: Collagen VI‐neural cell adhesion molecule 1 interaction. Neural Regeneration Research, 17(5), 1023–1033. 10.4103/1673-5374.324861 34558529 PMC8552870

[brb33174-bib-0049] Tambalo, S. , Peruzzotti‐Jametti, L. , Rigolio, R. , Fiorini, S. , Bontempi, P. , Mallucci, G. , Balzarotti, B. , Marmiroli, P. , Sbarbati, A. , Cavaletti, G. , Pluchino, S. , & Marzola, P. (2015). Functional magnetic resonance imaging of rats with experimental autoimmune encephalomyelitis reveals brain cortex remodeling. Journal of Neuroscience, 35(27), 10088–10100. 10.1523/JNEUROSCI.0540-15.2015 26157006 PMC4495237

[brb33174-bib-0050] Tang, S. , Qian, X. , Zhang, Y. , & Liu, Y. (2016). Treating low back pain resulted from lumbar degenerative instability using Chinese Tuina combined with core stability exercises: A randomized controlled trial. Complementary Therapies in Medicine, 25, 45–50. 10.1016/j.ctim.2016.01.001 27062947

[brb33174-bib-0051] Tracey, I. , & Mantyh, P. W. (2007). The cerebral signature for pain perception and its modulation. Neuron, 55(3), 377–391. 10.1016/j.neuron.2007.07.012 17678852

[brb33174-bib-0052] Wang, N. (2012). Effects of early application of Tuina treatment on quadriceps surface myoelectricity in patients after total knee arthroplasty: a randomized controlled trial. Zhong Xi Yi Jie He Xue Bao [Journal of Chinese Integrative Medicine], 10, 1247–1253. 10.3736/jcim20121108 23158943

[brb33174-bib-0053] Wu, J.‐J. , Lu, Y.‐C. , Hua, X.‐Y. , Ma, S.‐J. , Shan, C.‐L. , & Xu, J.‐G. (2018). Cortical remodeling after electroacupuncture therapy in peripheral nerve repairing model. Brain Research, 1690, 61–73. 10.1016/j.brainres.2018.04.009 29654733

[brb33174-bib-0054] Wu, J.‐J. , Lu, Y.‐C. , Hua, X.‐Y. , Ma, S.‐J. , & Xu, J.‐G. (2018). A longitudinal mapping study on cortical plasticity of peripheral nerve injury treated by direct anastomosis and electroacupuncture in rats. World Neurosurgery, 114, e267–282. 10.1016/j.wneu.2018.02.173 29524702

[brb33174-bib-0055] Xing, X.‐X. , Hua, X.‐Y. , Zheng, M.‐X. , Ma, Z.‐Z. , Huo, B.‐B. , Wu, J.‐J. , Ma, S.‐J. , Ma, J. , & Xu, J.‐G. (2020). Intra and inter: Alterations in functional brain resting‐state networks after peripheral nerve injury. Brain and Behavior, 10(9), e01747. 10.1002/brb3.1747 32657022 PMC7507705

[brb33174-bib-0056] Xing, X.‐X. , Zheng, M.‐X. , Hua, X.‐Y. , Ma, S.‐J. , Ma, Z.‐Z. , & Xu, J.‐G. (2021). Brain plasticity after peripheral nerve injury treatment with massage therapy based on resting‐state functional magnetic resonance imaging. Neural Regeneration Research, 16(2), 388–393. 10.4103/1673-5374.290912 32859803 PMC7896216

[brb33174-bib-0057] Xu, Y. , Tian, N.‐X. , Bai, Q.‐Y. , Chen, Q. , Sun, X.‐H. , & Wang, Y. (2019). Gait assessment of pain and analgesics: Comparison of the DigiGait and CatWalk gait imaging systems. Neuroscience Bulletin, 35(3), 401–418. 10.1007/s12264-018-00331-y 30659524 PMC6527535

[brb33174-bib-0058] Yamahachi, H. , Marik, S. A. , Mcmanus, J. N. J. , Denk, W. , & Gilbert, C. D. (2009). Rapid axonal sprouting and pruning accompany functional reorganization in primary visual cortex. Neuron, 64(5), 719–729. 10.1016/j.neuron.2009.11.026 20005827 PMC2818836

[brb33174-bib-0059] Yang, X. , Zhao, H. , & Wang, J. (2014). Chinese massage (Tuina) for the treatment of essential hypertension: A systematic review and meta‐analysis. Complementary Therapies in Medicine, 22(3), 541–548. 10.1016/j.ctim.2014.03.008 24906593

[brb33174-bib-0060] Yang, Y. , Wang, Z.‐H. , Jin, S. , Gao, D. , Liu, N. , Chen, S.‐P. , Zhang, S. , Liu, Q. , Liu, E. , Wang, X. , Liang, X. , Wei, P. , Li, X. , Li, Y. , Yue, C. , Li, H.‐L. , Wang, Ya‐L. , Wang, Q. , Ke, D. , … Wang, J.‐Z. (2016). Opposite monosynaptic scaling of BLP‐vCA1 inputs governs hopefulness‐ and helplessness‐modulated spatial learning and memory. Nature Communications, 7, 11935. 10.1038/ncomms11935 PMC4947155 27411738

[brb33174-bib-0061] Yuan, P. , & Raz, N. (2014). Prefrontal cortex and executive functions in healthy adults: A meta‐analysis of structural neuroimaging studies. Neuroscience and Biobehavioral Reviews, 42, 180–192. 10.1016/j.neubiorev.2014.02.005 24568942 PMC4011981

[brb33174-bib-0062] Zainul, Z. , Ma, B. , Koka, M. , Wilkerson, J. L. , Ortiz, Y. T. , Kerosuo, L. , & Chandran, V. (2022). Novel roles of phentolamine in protecting axon myelination, muscle atrophy, and functional recovery following nerve injury. Scientific Reports, 12(1), 3344. 10.1038/s41598-022-07253-w 35228612 PMC8885794

[brb33174-bib-0063] Zhang, J.‐P. , Shen, J. , Xiang, Y.‐T. , Xing, X.‐X. , Kang, B.‐X. , Zhao, C. , Wu, J.‐J. , Zheng, M.‐X. , Hua, Xu.‐Y. , Xiao, L.‐Bo. , & Xu, J.‐G. (2023). Modulation of brain network topological properties in knee osteoarthritis by electroacupuncture in rats. Journal of Pain Research, 16, 1595–1605. 10.2147/JPR.S406374 37220632 PMC10200108

[brb33174-bib-0064] Zhang, W.‐T. , Jin, Z. , Cui, G.‐H. , Zhang, K.‐L. , Zhang, L. , Zeng, Y.‐W. , Luo, F. , Chen, A. C. N. , & Han, J.‐S. (2003). Relations between brain network activation and analgesic effect induced by low vs. high frequency electrical acupoint stimulation in different subjects: A functional magnetic resonance imaging study. Brain Research, 982(2), 168–178. 10.1016/s0006-8993(03)02983-4 12915252

[brb33174-bib-0065] Zhang, Y. , He, X. , Hu, S. , Hu, S. , He, F. , Shen, Y. , Zhao, F. , Zhang, Q. , Liu, T. , & Wang, C. (2020). Efficacy and safety of massage in the treatment of post‐stroke insomnia: A protocol for systematic review and meta‐analysis. Medicine, 99(51), e23598. 10.1097/MD.0000000000023598 33371092 PMC7748325

[brb33174-bib-0066] Zhou, X. , Kong, L. , Ren, J. , Song, P. , Wu, Z. , He, T. , Lv, Z. , Zhang, S. , Sun, W. , Zhang, J. , Cai, J. , Zhu, Q. , & Fang, M. (2022). Effect of traditional Chinese exercise combined with massage on pain and disability in patients with lumbar disc herniation: A multi‐center, randomized, controlled, assessor‐blinded clinical trial. Frontiers in Neurology, 13, 952346. 10.3389/fneur.2022.952346 36158965 PMC9490580

[brb33174-bib-0067] Zitnik, G. A. , Clark, B. D. , & Waterhouse, B. D. (2014). Effects of intracerebroventricular corticotropin releasing factor on sensory‐evoked responses in the rat visual thalamus. Brain Research, 1561, 35–47. 10.1016/j.brainres.2014.02.048 24661913 PMC4019997

[brb33174-bib-0068] Zubieta, J.‐K. , Smith, Y. R. , Bueller, J. A. , Xu, Y. , Kilbourn, M. R. , Jewett, D. M. , Meyer, C. R. , Koeppe, R. A. , & Stohler, C. S. (2001). Regional mu opioid receptor regulation of sensory and affective dimensions of pain. Science, 293(5528), 311–315. 10.1126/science.1060952 11452128

